# Levels of polycyclic aromatic hydrocarbon in the soil around typical automobile repair workshops in Nigeria

**DOI:** 10.12688/f1000research.134682.1

**Published:** 2023-07-20

**Authors:** Olusola Adedayo Adesina, Oluwatomi Atinuke Fakayode, Mayowa Adeoye Lala, Abiodun John Adewale, Jacob Ademola Sonibare

**Affiliations:** 1 Department of Chemical and Petroleum Engineering, Afebabalola University, Ado Ekiti, Ekiti, Nigeria; 2 Department of Chemical Engineering, Landmark University, Omu-Aran, Kwara, Nigeria; 3 Department of Chemical Engineering, Obafemi Awolowo University, Ife-Ile, Osun, Nigeria

**Keywords:** PAHs, ambient air, automobile workshop, Nigeria

## Abstract

**Background**: This study determined the levels of polycyclic aromatic hydrocarbons (PAHs) in the soil around typical automobile repair workshops in Nigeria. Risk assessment associated with human contact with the soil was carried out using hazard quotient (HQ) and incremental life cancer risk (ILCR) from human unconscious ingestion and dermal contact with the soil.

**Methods**: Soil samples were obtained at different automobile workshops in Ado-Ekiti, Western Nigeria. The PAHS in the samples were extracted using dichloromethane and extracts were cleaned up using silica-alumina gel open column chromatography. Analysis of PAHs in the soil was done using a gas chromatograph coupled to a mass selective detector operated on electron ionization mode.

**Results**: The results showed the mean PAHs concentration at the sampling locations ranged from 5.58 – 6.4 *μg/g* and the mean ∑ carcinogenic PAHs was 58.4 *μg/g*, equivalent to 59.39 % of total PAHs observed. The mean Toxicity equivalence (TEQs) ranged from 0.02 - 6.680 *μg/g.* Benzo (a) pyrene and dibenzo(a,h)anthracene have the highest toxicity equivalent. The total ILCRs from accidental ingestion and dermal contact in adults were *1 ×10^(-3) and 9.8 ×10^(-5)* , for adults and children respectively; both are higher than the permissible limit stipulated by the World Health Organization.The HQs obtained are several folds higher than 1. This implies high carcinogenic and non-carcinogenic risks for children and adults.

**Conclusions**: The study revealed the levels of PAHs and also revealed the risks associated with human contact with the soil around automobile repair workshops.

## Introduction

Indiscriminate discharging of used engine oil into the surroundings, especially around automobile workshops, remains a major problem in most parts of Nigeria and this constantly affects the ecosystem ( Odjegba and Sadiq, 2002; Emoyan *et al*., 2020). The soil around automobile workshops is usually contaminated with spent engine oil which contains heavy metals and polycyclic aromatic compounds (PAHs) and other pollutants. PAHs constitute two or more fused aromatic rings solely made from carbon and hydrogen ( Arey and Atkinson, 2003). PAHs are emitted from the exhaust of automobiles as well as by-products from petroleum ranging from lubricating oil, gasoline, diesel, and several others put to constant use in automobile workshops, and these have high chances of spilling into and degrading the soil ( Kidman and Boehlecke, 2011; Muze *et al*., 2020).

PAHs, which are the products of incomplete combustion, are transported into the crankcase and concentrated in lubricating oil in the engine of the car ( Pruell and Quinn, 1988; Akintunde *et al*., 2015). Additionally, emissions from the exhausts of the cars being repaired at these workshops can be washed down through rain to the soil around the workshop. Attention has been drawn to the health risks associated with human contact with soil contaminated with PAHs ( Wcisło, 1998; Adeniyi and Afolabi, 2002; Dong and Lee, 2009; Kwon and Choi, 2014). PAHs are found to have a negative effect on human health as they can lead to cancer, as well as having a mutagenic and teratogenic effect ( Boström *et al.,* 2002).

The presence of PAHs in the spent engine oils disposed in the environment during maintenance poses a serious threat to the environment ( Ololade, 2014). The lack of required knowledge on proper handling of these hydrocarbon products during repairs and vehicle servicing in automobile workshops is a major problem, especially in Nigeria ( Sharifi *et al*., 2007). Akintunde *et al*. (2015) studied the reproductive effects of used engine oil on male rats, and their results showed used engine oil has the potential to hamper male rats' germ cell development and also affect other testicular activities in producing viable spermatozoa. Their study also concluded that soil contaminated with spent engine oil poses a great reproductive risk to humans in areas where there is high exposure. The Agency of Toxic Substances and Diseases Registry rates PAHs as ninth on the list of compounds dangerous to human health. Organisms are also affected by the toxic effect of PAHs as they affect the functioning process of cellular membranes ( Abdel-Shafy and Mansour, 2016). Clients and workers at automobile workshops are exposed to PAHs through contact with the soil and ambient around the workshop.

Few or no studies are available on the level of PAHs around automobile repair workshops, especially in Sub-Saharan Africa countries such as Nigeria. Hence this study focuses on determining the levels of PAHs around automobile repair workshops, this is with a view to determining the risk associated with human contact with these pollutants.

## Methods

### Study area

The study area locations are shown in Table 1. In order to assess the implication of automobile repair workshops’ activities on the soil levels of PAHs, soil samples were taken from five different local automobile repair workshops (locations A-E) in Ado-Ekiti, Southwestern Nigeria. Ado-Ekiti is the capital of Ekiti state in Southwestern Nigeria and is a city on 7° 37′ 15.9996″ N and 5° 13′ 17.0004″ E.

**Table 1.** Sampling locations.

Label	Latitude	Longitude
A	7° 36′ 27″ N	5° 12′ 52″ E
B	7° 37′ 1″ N	5° 12′ 44″ E
C	7° 36′ 48″ N	5° 12′ 36″ E
D	7° 36′ 30″ N	5° 12′ 50″ E
E	7° 36′ 35″ N	5° 15′ 17″ E

### Soil sampling of PAHs

The top layer of soil samples (0–15 cm) was collected around the automobile workshops. The samples were wrapped in foil papers after collection and transported to the laboratory for analysis. At the laboratory, the impurities were then removed by hand picking, and then sieved through a 2 mm sieve shaker. The samples were then oven dried using a universal drier at 80 ^o^C to constant mass, and they were then kept in aluminum foil bags and stored at -20 °C in the freezer.

### Sample pretreatment

The soil samples were initially spiked with recovery standards of PAHs to monitor the integrity of the treatment. The sample was then extracted with dichloromethane (DCM) using a Soxhlet extractor at 30°C for 8 hrs to enable PAHs trapped on the PUF disk and soil to dissolve in DCM. A clean-up procedure was done to remove unwanted compounds in the matrix by using silica-alumina column chromatography. The column was prepared by adding about 10 to 15 mm plug of glass wool to a chromatograph column and stuffing it down using a glass rod, then alumina and silica were added (1:2). The column packing was partially deactivated using a methanol-DCM solution (1:3) for better recovery. The sample extract was decanted into the column and eluted with 40 mL 1:1, DCM: hexane ( Adesina *et al*., 2018). The extracted samples were then concentrated using a rotary evaporator (Büchi ^®^), to gently remove the solvent from the extract in order to bring down the volume of extract to 5ml **.** The resultant extracts were later analyzed for PAHs.

### PAHs analysis

Quantification of PAHs present in the sample was carried out using gas chromatography (GC) (Agilent 7890) with a mass detector (Agilent 5975) operated in a selected ion monitoring mode and using electron impact ionization (EI). The chromatographic column dimensions are 30 m × 0.25 mm, and the internal diameter of 0.25 μm film thickness. The temperature program for the analysis was set as 90°C (1.0 min), 30°C/min, 250°C, 4°C/min, and 330°C (5 min). Determination of the concentration of PAHs was done using the internal standard method. The internal standards are naphthalene-d _8_, acenaphthene-d _10_, phenanthrene-d _10_, chrysene-d _12_, and perylene-d _12_, used to quantify the amount of PAHs in the extract.

### Quality assurance/quality control (QA/QC)

Apart from normal samples, field and laboratory blanks were also taken and treated the same way as the samples to ensure high integrity of the data. Soil blank samples were taken at locations far from the workshops, that have not been contaminated by automobile repair activities. Determination of instrument detection limits (IDLs) and method detection limits (MDLs) followed Norlock *et al.* (2011) procedure. Standard solutions were prepared for PAHs and six surrogates which were analyzed four times in the SIM mode using GC-MS. The PAH concentrations in each calibration solution were recalculated from the regression equation obtained using all six calibration standards. The average of the four replicates with the lowest detectable concentration was taken as the IDL. MDLs were calculated by multiplying the standard deviation of the replicates by the one-side t statistic at the 99%. Before the extraction, 20 ng of phenanthrene d10 recovery standard (RS) was used to spike the sample and the recovery range of the PAH was between 80% and 90%. Concentrations of PAHs in the field blanks were below the detection limit for all targeted compounds and no blank correction was carried out.

### Health implications

**Toxic equivalent**

The potential toxicity of the PAHs is calculated by multiplying the individual concentration, *C*, with the toxicity equivalence factor (TEF) ( eq. i) ( Van den Berg *et al.,* 2006).

$$\text{Toxic equivalecy}\;\left(\mathrm{TEQ}\right)=C\times \mathrm{TEF}$$ (i)

ILCR from ingestion, inhalation, and dermal contact with PAHs contaminated soil are calculated using eq. ii, iii, iv, respectively. Also, the non-carcinogenic associated risk is assessed using the hazard quotient index (HQ) which is the ratio of the estimated to the reference dose using eq. v ( USEPA, 1991, 2011).

$${\text{ILCR}}_{\text{ingestion}}=\frac{C\times \left({\mathrm{CSF}}_{\mathrm{ing}}\times \sqrt[{3}]{\left(\frac{\mathrm{BW}}{70}\right)}\times {\mathrm{IR}}_{\mathrm{ing}}\times \mathrm{EF}\times \mathrm{ED}\right)}{\mathrm{BW}\times \mathrm{AT}\times 10^{6}}$$ (ii)

$${\text{ILCR}}_{\text{inhalation}}=\frac{C\times \left({\mathrm{CSF}}_{\mathrm{inh}}\times \sqrt[{3}]{\left(\frac{\mathrm{BW}}{70}\right)}\times {\mathrm{IR}}_{\mathrm{inh}}\times \mathrm{EF}\times \mathrm{ED}\right)}{\mathrm{BW}\times \mathrm{AT}\times \mathrm{PEF}}$$ (iii)

$${\text{ILCR}}_{\text{dermal}}=C\times \left(\frac{{\mathrm{CSF}}_{\text{derm}}\times \left(\sqrt[{3}]{\left(\frac{\mathrm{BW}}{70}\right)}\times \mathrm{SA}\times \mathrm{AF}\times \mathrm{ABS}\times \mathrm{EF}\times \mathrm{ED}\right)}{\mathrm{BW}\times \mathrm{AT}\times 10^{6}}\right)$$ (iv)

$$\mathrm{HQ}=\frac{\mathrm{EDI}}{\mathrm{RfD}}$$ (v)

$$\mathrm{EDI}=\frac{\mathrm{CS}\times \mathrm{IR}\times \mathrm{ED}\times \mathrm{EF}\times \mathrm{CF}}{\mathrm{AT}\times \mathrm{BW}}$$

*C* is the PAH concentration in the soil solid residue (mg kg ^−1^). ${\mathrm{IR}}_{\mathrm{ing}}$ is the soil ingestion rate (100 mg d ^−1^ for adults and 200mg d ^−1^for children). ${\mathrm{IR}}_{\mathrm{inh}}$ is the inhalation rate (20 m ^3^/day was assumed for adults while 9.6 m ^3^/day was assumed for children). Table 2 shows the exposure parameters and factors used for the study.

**Table 2.** Exposure parameters and factors used for the study.

Exposure factors	Adult	Child	Reference
Ingestion rate (mg/day)	100	200	( USEPA, 2011)
Exposed skin area, SA (cm ^2^)	5700	2800	( USEPA, 2011)
Skin adherence factor, AF _soil_ (mg/cm ^2^)	0.07	0.2	( USEPA, 2011)
Exposure frequency, EF (days/year)	365	365	( Kumar *et al*., 2013)
Exposure duration, ED (year)	24	6	( USEPA, 2011)
Body weight, BW (kg)	60	18	( Adesina *et al.*, 2023)
Averaging time, AT (days) - (70 years × 365 days/year)	25550	25550	( Ferreira-Baptista and Miguel, 2005)
Dermal adsorption fraction (ABS)	0.13	0.13	( USEPA, 2011)
Inhalation rate (m ^3^/day)	20	10	( Soltani *et al.*, 2015)
Particulate emission factor (m ^3^/kg)	1.36 × 10 ^9^	1.36 × 10 ^9^	( USEPA, 2011)
CSF ingestion (mg/kg/day)	7.3	7.3	( Peng *et al.*, 2011)
CSF inhalation (mg/kg/day)	3.85	3.85	( Peng *et al.*, 2011)
CSF dermal (mg/kg/day)	25	25	( Peng *et al.*, 2011)

## Results and discussion

In this study, 16 different USEPA priority PAHs were analyzed in soil samples: naphthalene (Naph), acenaphthylene (Acy), acenaphthene (Ace), fluorene (Fln), phenanthrene (Phe), pyrene (Pyr), fluoranthene (Flt), anthracene (Ant), benzo [e] pyrene (BeP), benzo [a] pyrene (BaP), benzo [b] fluoranthene (BbF), benzo [a] anthracene (BaA), chrysene (CHR), benzo [k] fluoranthene (BkF), indeno[1,2,3-cd] pyrene (InP), and dibenzo [a,h] anthracene (DAh). Table 3 shows the concentrations of PAHs at different automobile workshops. The results showed BbF and Bkf have the highest concentrations. The concentration of BbF ranged from 4.0 – 31.03 μg/g with a mean concentration of 17.30 μg/g . BkF’s concentration ranged from 4.12-27.92 μg/g with a mean concentration of 17.53. These two compounds contribute 36 % of the total PAHs found in the soil. A high concentration of this compound implies that the source of the PAHs contamination of the soil is largely from petroleum products such as spent engine oil. Another compound with a high concentration is Fln, with a range of 3.96 – 18.13 μg/g and a mean concentration of 10.81 μg/g , which is 11 % of the total PAHs. Fln is one of the Middle Molecular Weight PAHs formed by the combustion of petroleum products such as emission from the exhaust of the vehicle. This could be the reason for the high concentration of this compound around the soil analyzed. BaP concentration is usually used as an indicator of PAH carcinogenic activity due to its stability. The result obtained showed a BaP mean concentration of 5.39 μg/g with a range of 0.26-11.5 μg/g , which shows carcinogenic activities. The mean concentration of other carcinogenic PAHs observed in the soil is benzo [a] anthracene (2.45 μg/g) , chrysene (3.24 μg/g) , benzo [b] fluoranthene (17.30 μg/g) benzo [k] fluoranthene ( 17.53μg/g ) and indeno (1, 2, 3-cd) pyrene (5.83 μg/g) . Generally, the result showed the mean ∑ carcinogenic PAHs observed is 58.4 μg/g which is equivalent to 59.39% of total PAHs.

**Table 3.** Mean concentration and TEQs of different PAH compounds in the soil.

PAHs	Mean	Std	Min	Max	% of Total	TEQ
Naph	1.65	0.62	0.72	2.03	2	0.002
Acy	3.36	2.39	1.05	6.5	3	0.003
Acen	5.36	4.52	2.27	12.01	5	0.005
Fln	10.81	7.84	3.96	18.13	11	0.011
Phe	3.55	1.33	2	4.92	4	0.004
Ant	3.38	2.42	0.98	6.03	3	0.034
Flt	5.17	3.14	2.29	8.93	5	0.005
Pyr	6.67	6.88	1.18	16.05	7	0.007
BbF	17.30	14.06	4	31.03	18	1.730
BkF	17.53	10.43	4.01	27.92	18	1.753
CHR	3.24	1.71	1.54	4.84	3	0.032
BaP	5.39	5.69	0.26	11.15	5	5.390
BaA	2.45	2.02	0.62	4.9	2	0.245
IcP	5.83	4.58	1.97	12.04	6	0.583
DhA	6.68	2.50	5.04	10.38	7	6.680
BgP	0	0.00	0	0	0	0.000
∑ PAHS	98.34					16.49
∑ Carcin-PAHs	58.4	9.4			59.39%	

### Distribution of PAHs based on aromatic rings and molecular weight

**Figure 1.**
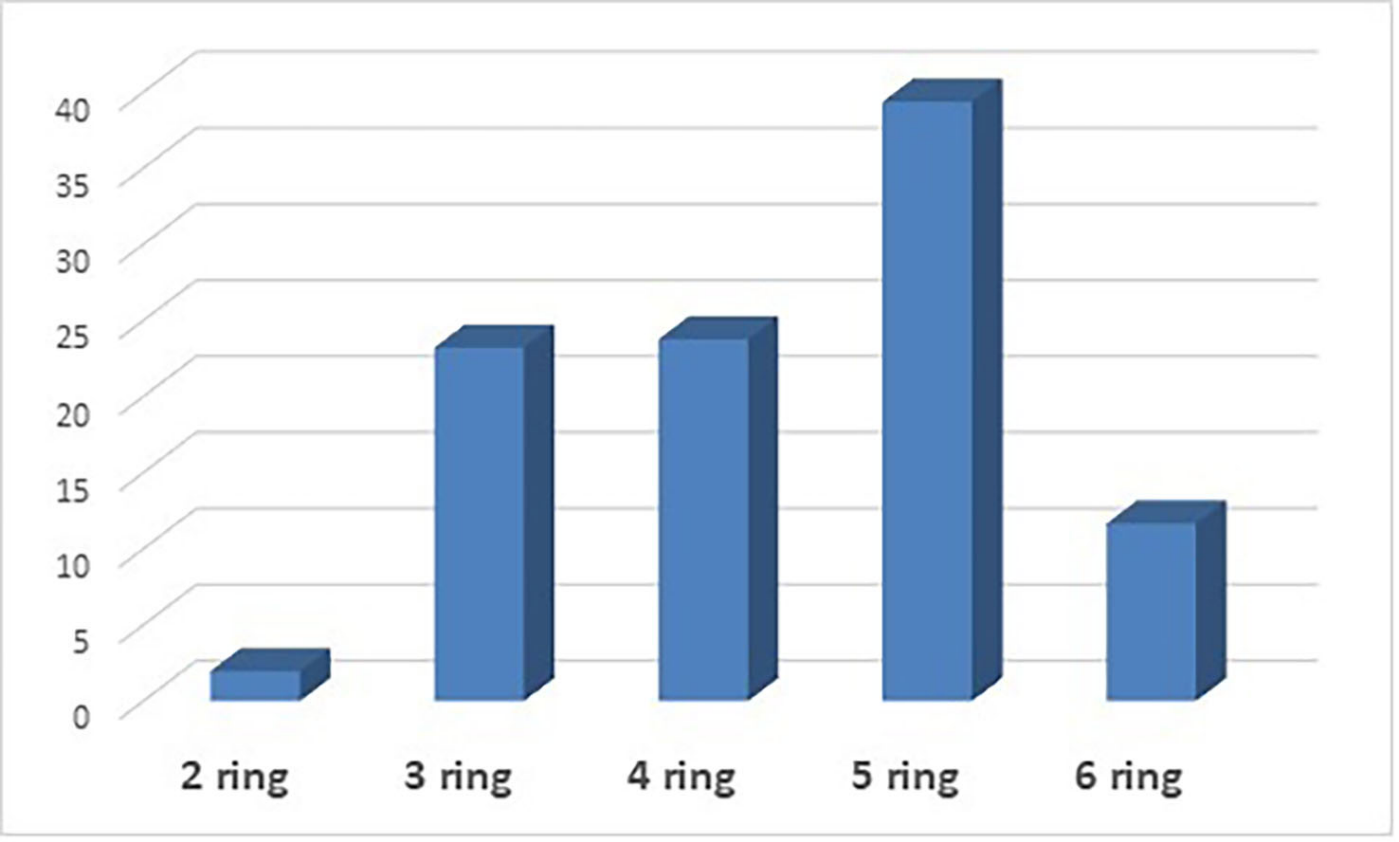
Distribution of PAHs based on the number of aromatic rings.

**Figure 2.**
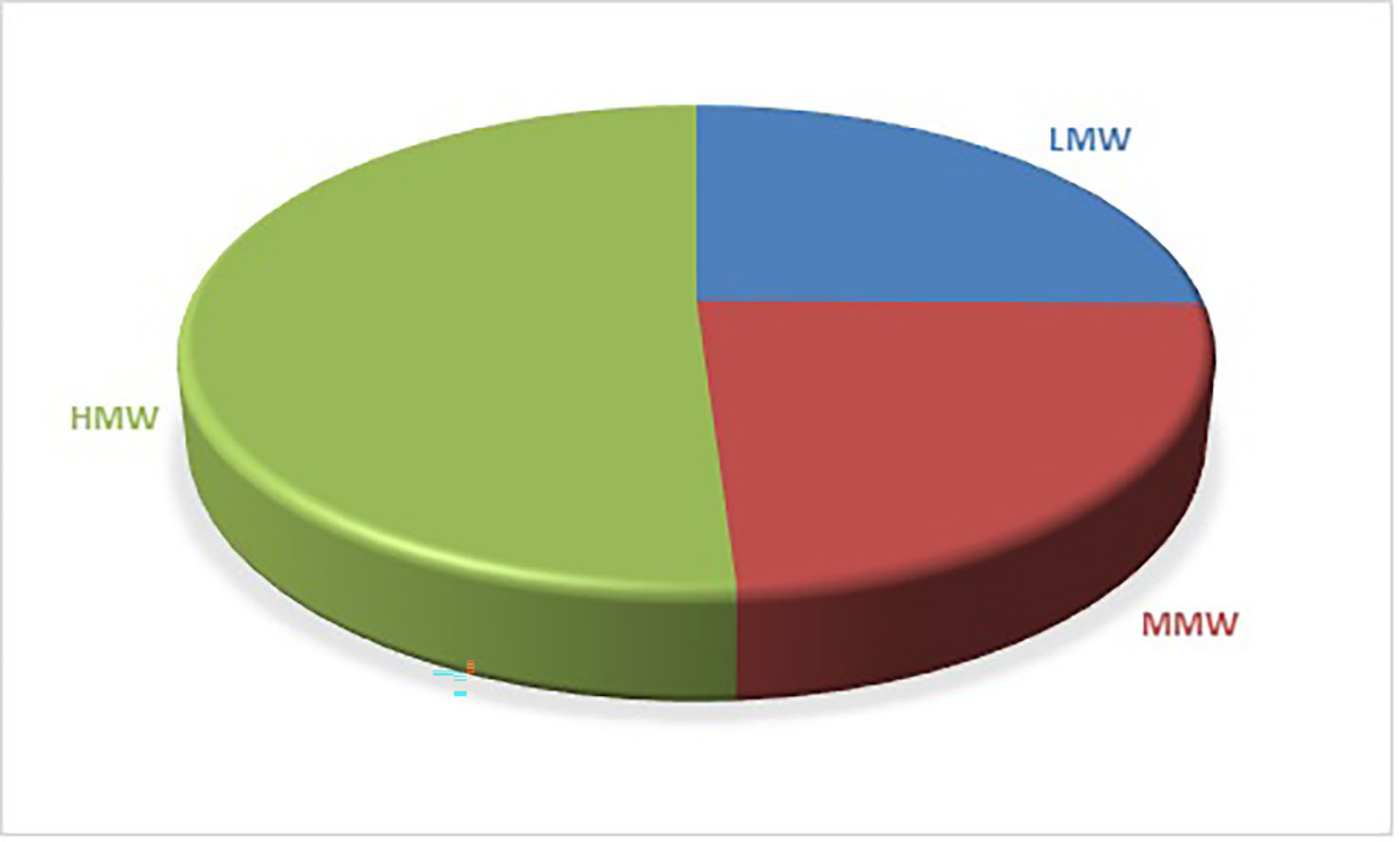
Distribution of PAHs based on molecular weight.

PAHs are also classified based on the number of aromatic rings in each compound. The following ring compounds were present in this analysis: 2-ring PAHs (naphthalene), 3-ring PAHs (acenaphthylene, acenaphthene, fluorene, anthracene, and phenanthrene), 4-ring PAHs (pyrene, fluoranthene, chrysene, and benzo [a]anthracene), 5-rings PAHs (benzo [b] fluoranthene, benzo [a] pyrene, benzo [e] pyrenes, dibenzo [a,h] anthracene, and benzo [k]fluoranthene) and 6-rings PAHs (indeno [1,2,3-cd]pyrene). Figure 1 shows the distribution of PAHs based on the number of rings. 5-ring PAHs have the highest percentage at 39.37%, followed by 4-ring PAHs at 23.77%. 3-ring PAHs have a percentage contribution of 23.23%, 6-ring PAHs have a percentage contribution of 11.67%, and 2-ring PAHs have the lowest contribution at 1.97%. PAHs classification can also be by molecular weight: low molecular weight (LMW), consisting of 2- and 3-ring PAHs, middle molecular weight (MMW) consisting of 4-ring PAHs, and high molecular weight (HMW), consisting of 5- and 6-ring PAHs. Based on the results in Figure 2, LMW accounts for 25.2% of the total PAHs, MMW accounts for 23.77% and HMW accounts for the highest percentage of 51.03%.

### Health implications of PAHs in the soil sample

The TEQ approach is used to determine the toxic potency of complex mixtures, the ILCR is used to determine the probability of developing cancer as the result of exposure to a specific carcinogen, and the hazard quotient is the ratio of the potential exposure to a substance and the level at which no adverse effects are expected. Table 3 shows the TEQ of the PAHs in all the soil samples analyzed. DhA has the highest toxicity with a mean value of 6.68 μg/g. BaP, which is usually used as an indicator of PAHs contamination, has a toxicity of 5.4 μg/g. Other compounds with high TEQ are BbF and BkF with mean values of 1.73 and 1.75 μg/g, respectively.

The ILCR is used to assess the probability of developing cancer by having contact with soil from the automobile workshop. Table 4 shows the ILCR and HQ values obtained from the study. The ILCR values from accidental ingestion of soil from these workshops are $3.9\times 10^{-4}$ and $4.4\times 10^{-4}$ for adults and children respectively, while ILCR values for dermal contact with the soil are $6.9\times 10^{-4}$ and $5.4\times 10^{-4}.$ The value is higher than the permissible limit of $10^{-6}$ stipulated by the World Health Organization. This implies that, with exposure to this soil for a particular period, there is a probability of developing cancer. However, the ILCR values for accidental inhalation of the soil are $3.2\times 10^{-8}$ and $8\times 10^{-9}$ , for adults and children respectively. This indicates the chances of developing cancer from inhalation of this soil are slim. The combination of ILCR from accidental ingestion, inhalation and dermal contact gives $1\times 10^{-3}$ and $9.8\times 10^{-5}$ , for adults and children respectively. These values are higher than the permissible limit.

**Table 4.** ILCR and hazard quotient.

	Adult	Children
**ILCR**_**ingestion**_	$3.9\times 10^{-4}$	$4.4\times 10^{-4}$
**ILCR**_**inhalation**_	$3.2\times 10^{-8}$	$8\times 10^{-9}$
**ILCR**_**dermal**_	$6.9\times 10^{-4}$	$5.4\times 10^{-4}$
**ILCR**_**Total**_	$1\times 10^{-3}$	$9.8\times 10^{-5}$
**HQ**	28097.14	46828.57

Table 4 also shows the hazard quotient, which is several folds higher than 1, the permissible limit, which implies that there is great non-carcinogenic risk associated with exposure to this soil.

## Conclusion

This work studied the levels of polycyclic aromatic hydrocarbons in the soil around typical automobile repair workshops in Nigeria. The study also assessed the risks associated with human contact with the soil. The results showed the mean PAHs concentration at the sampling locations ranged from 5.58 – 6.4 μg/g . The total toxicity equivalents at various locations range from 8.57 to 16.6 μg/g . The value of summation of ILCR and HQs from ingestion, inhalation, and dermal contact with PAHs contaminated soil is higher than the permissible limit stipulated by the World Health Organization. This study revealed the risk associated with human contact with soil around automobile workshops in Nigeria.

## Data Availability

Zenodo: Levels of polycyclic aromatic hydrocarbon in the soil around typical automobile repair workshops in Nigeria. https://doi.org/10.5281/zenodo.7939076 ( Adesina *et al*., 2023). Data are available under the terms of the Creative Commons Attribution 4.0 International license (CC-BY 4.0).

## References

[ref1] Abdel-Shafy HI Mansour MS : A review on polycyclic aromatic hydrocarbons: Source, environmental impact, effect on human health and remediation. *Egypt. J. Pet.* 2016;25(1):107–123. 10.1016/j.ejpe.2015.03.011

[ref2] Adeniyi AA Afolabi JA : Determination of total petroleum hydrocarbons and heavy metals in soils within the vicinity of facilities handling refined petroleum products in Lagos Metropolis. *Environ. Int.* 2002;28(1-2):79–82. 10.1016/S0160-4120(02)00007-7 12046957

[ref3] Adesina OA Sonibare JA Diagboya PN : Spatiotemporal distributions of polycyclic aromatic hydrocarbons close to a typical medical waste incinerator. *Environ. Sci. Pollut. Res.* 2018;25(1):274–282. 10.1007/s11356-017-0335-1 29032527

[ref4] Adesina OA Fakayode OA Lala MA : LEVELS OF POLYCYCLIC AROMATIC HYDROCARBON IN THE SOIL AROUND TYPICAL AUTOMOBILE REPAIR WORKSHOPS IN NIGERIA.[Dataset]. *Zenodo.* 2023. 10.5281/zenodo.8034784 PMC11342030 39183733

[ref5] Akintunde WO Olugbenga OA Olufemi OO : Some Adverse Effects of Used Engine Oil (Common Waste Pollutant) On Reproduction of Male Sprague Dawley Rats. *Med. Sci.* 2015;3(1):46–51. 10.3889/oamjms.2015.035 27275195 PMC4877787

[ref6] Arey J Atkinson R : Photochemical reactions of PAH in the atmosphere. Douben PET , editor. *PAHs: An ecotoxicological perspective.* 2003;4(47–63). 10.1002/0470867132.ch4

[ref7] Boström CE Gerde P Hanberg A : Cancer risk assessment, indicators and guidelines for polycyclic aromatic hydrocarbons in ambient air. *Environ. Health Perspect.* 2002;110(3):451–489. 10.1289/ehp.02110s3451 Reference Source 12060843 PMC1241197

[ref8] Dong TT Lee BK : Characteristics, toxicity, and source apportionment of polycyclic aromatic hydrocarbons (PAHs) in road dust of Ulsan, Korea. *Chemosphere.* 2009;74:1245–1253. 10.1016/j.chemosphere.2008.11.035 19103459

[ref9] Emoyan OO Onocha EO Tesi GO : Concentration assessment and source evaluation of 16 priority polycyclic aromatic hydrocarbons in soils from selected vehicle-parks in southern Nigeria. *Scientific Africa.* 2020;7(2):e00296–e00213. 10.1016/j.sciaf.2020.e00296

[ref22] Ferreira-Baptista L De Miguel E : Geochemistry and Risk Assessment of Street Dust in Luanda, Angola: A Tropical Urban Environment. *Atmos. Environ.* 2005;39(25):4501–4512. 10.1016/j.atmosenv.2005.03.026.18

[ref10] Kidman RL Boehlecke R : Evaluating petroleum hydrocarbon-contaminated soil. *WM 20 conference.* Phoenix;2011.

[ref21] Kumar B Verma VK Kumar S : Probabilistic Health Risk Assessment of PolycyclicAromatic Hydrocarbons and Polychlorinated Biphenyls in Urban Soils from a Tropical City of India. *J. Environ. Sci. Health A Tox Hazard Subst. Environ. Eng.* 2013;48(10):1253–1263. 10.1080/10934529.2013.776894.(18)(PDF) 23647116

[ref11] Kwon HO Choi SD : Polycyclic aromatic hydrocarbons (PAHs) in soils from a multi-industrial city, South Korea. *Sci. Total Environ.* 2014;470-471:1494–1501. 10.1016/j.scitotenv.2013.08.031 24011990

[ref12] Muze NE Opara AI Ibe FC : Assessment of the geo-environmental effects of activities of auto-mechanic workshops at Alaoji Aba and Elekahia Port Harcourt, Niger Delta, Nigeria. *Environ. Anal. Health Toxicol.* 2020;35(2):1–12. 10.5620/eaht.2020005 PMC7374190 32693557

[ref25] Norlock FM Jang J Zou Q : Large-Volume Injection PTV-GC-MS Analysis of Polycyclic Aromatic Hydrocarbons in Air and Se diment Samples. *J. Air Waste Manage. Assoc.* 2011;52(1):19–26. 10.1080/10473289.2002.10470752 15152661

[ref13] Odjegba VJ Sadiq AO : Effects of spent engine oil on the growth parameters, chlorophyll and protein levels of *Amaranthus hybridus* L. *Environmentalist.* 2002;22(1):23–28. 10.1023/A:1014515924037

[ref16] Ololade IA : An assessment of heavy metal contamination in soils within auto-mechanic workshops using enrichment and contamination factors with geo – accumulation Indexes. *J. Environ. Prot.* 2014;05:970–982. 10.4236/jep.2014.511098

[ref24] Peng Chen W Liao X : Polycyclic Aromatic Hydrocarbons in Urban Soils of Beijing: status, Sources, Distribution and Potential Risk. *Environ. Pollut. (Barking, Essex: 1987).* 2011;159(3):802–808. 10.1016/j.envpol.2010.11.003 21159413

[ref14] Pruell RJ Quinn JQ : Accumulation of polycyclic aromatic hydrocarbons in crankcase oil. *Environ. Pollut.* 1988;49:(2):89–97. 10.1016/0269-7491(88)90242-4 15092665

[ref17] Sharifi M Sadeghi Y Akbarpour M : Germination and growth of six plant species on contaminated soil with spent oil. *Int. J. Environ. Sci. Technol.* 2007;4(4):463–470. 10.1007/BF03325982

[ref23] Soltani N Keshavarzi B Moore F : Ecological and Human Health Hazards of Heavy Metals and Polycyclic Aromatic Hydrocarbons (PAHs) inRoad Dust of Isfahan Metropolis, Iran. *Sci. Total Environ.* 2015;505:712–723. 10.1016/j.scitotenv.2014.09.097C 25461074

[ref19] United States Environmental Protection Agency, USEPA: *Exposure factors handbook (Final).* Washington:2011.

[ref20] USEPA: Guidelines for developmental toxicity risk assessment. 1991. Reference Source

[ref15] Wcisło E : Soil contamination with polycyclic aromatic hydrocarbons (PAHs) in Poland – a review. *Pol. J. Environ. Stud.* 1998;7(5):267–272.

[ref18] Van den Berg M Linda SB Michael D : The 2005 World Health Organization re-evaluation of human and mammalian toxic equivalency factors for dioxins and dioxin-like compounds. *Toxicol. Sci.* 2006;93:223–241. 10.1093/toxsci/kfl055 16829543 PMC2290740

