# Supplementary material for: Levels of polycyclic aromatic hydrocarbon in the soil around typical automobile repair workshops in Nigeria
Source: F1000Res. 2024 Feb 12;12:Chem Inf Sci-861. Originally published 2023 Jul 20. [Version 3] doi: 10.12688/f1000research.134682.3 (PMC11342030; doi:10.12688/f1000research.134682.3)
Supplement: Peer review history

# Reviewer report: Reviewer response for version 3

**DOI:** 10.5256/f1000research.161209.r315376

**Published:** 2024-08-22

**Authors:** Loai Aljerf

**Affiliations:** 1 Faculty of Pharmacy, Al-Sham Private University, Damascus, Damascus Governorate, Syria

General Assessment:

 The manuscript, "Levels of polycyclic aromatic hydrocarbon in the soil around typical automobile repair workshops in Nigeria," presents a valuable investigation into the contamination of soil with polycyclic aromatic hydrocarbons (PAHs) around automobile repair workshops in Nigeria. The study's objectives, methodology, and results are well-structured, and the manuscript provides a comprehensive overview of the problem, highlighting the risks associated with human contact with PAH-contaminated soil.

 Strengths:

 1) Relevance: 1- The study addresses a critical environmental and health issue in Nigeria, where the indiscriminate disposal of used engine oil and other petroleum products is a significant concern. 2- The study's focus on PAH levels in soil around automobile repair workshops in Nigeria is timely and significant, given the potential health risks associated with PAH exposure. 3- The study addresses a critical environmental issue, investigating the levels of PAHs in soil around automobile repair workshops in Nigeria. The findings have important implications for human health and environmental protection.

 2) Methodology: 1- The authors employed a robust methodology, including dichloromethane extraction, silica-alumina gel open column chromatography, and gas chromatography-mass spectrometry (GC-MS) analysis, to determine the levels of PAHs in soil samples. 2- The authors have provided a detailed description of their sampling, extraction, and analytical procedures, which demonstrates a good understanding of the research design and protocols.

 3) Comprehensive analysis: The authors have conducted a thorough analysis of 16 different USEPA priority PAHs in soil samples, providing a detailed understanding of the distribution and concentration of these pollutants.

 4) Inclusion of quality control measures: The authors have incorporated quality assurance and quality control measures, such as the use of field and laboratory blanks, recovery standards, and instrument detection limits, to ensure the integrity of their data.

 5) Risk Assessment: The study's risk assessment, which included the calculation of hazard quotients (HQs) and incremental life cancer risks (ILCRs), provides valuable insights into the potential health risks associated with human contact with PAH-contaminated soil.

 Weaknesses:

1. Literature Review: 1- Although the authors provide a good overview of the problem, the literature review could be more comprehensive, covering recent studies on PAH contamination in soil and its health risks. 2- Lack of contextualization: The study would benefit from a more extensive introduction, providing context on the environmental and health impacts of PAHs, as well as the current state of knowledge on this topic in Nigeria.
2. Limited study area: The study is limited to a single city in Southwestern Nigeria, which may not be representative of the entire country or other regions. Future studies should consider expanding the study area to increase generalizability.
3. Methodology: The authors employed a robust methodology, including the use of the Toxic Equivalence (TEQ) approach, ILCR, and HQ to assess the toxicity and carcinogenic potential of PAHs in the soil samples.
4. Sampling Strategy: 1- The manuscript does not provide sufficient information on the sampling strategy, including the number of samples, sampling locations, and sampling depth. This information is essential to understand the representativeness of the results. 2- The sampling strategy, although described in detail, may benefit from a more systematic approach, such as a stratified random sampling design, to minimize bias and ensure a more representative sample.
5. Analytical methods: Despite, the authors have used a well-established analytical method (GC-MS), it would be beneficial to provide more information on the method's performance, such as the limit of detection, limit of quantitation, and precision.
6. Quality Control and Assurance: The authors should provide more details on the quality control and assurance measures taken during the analysis, such as the use of certified reference materials, blanks, and duplicates.
7. Data Presentation: 1- The results could be presented more effectively, with clearer tables and figures that facilitate the comparison of results across different sampling locations.  
8. Health implications: The calculation of TEQ and ILCR is a strength of the study, but the authors could further explore the health implications of their findings, including a discussion of the potential risks to local residents and workers in the automobile repair industry.
9. The study would benefit from a more comprehensive comparison with existing literature on PAH levels in soil and their health implications, which would help to contextualize the findings and identify knowledge gaps.
10. Discussion and Conclusion: The discussion and conclusion sections could be more comprehensive, highlighting the implications of the findings and providing recommendations for policymakers, automobile workshop owners, and the general public.

Aspects Not Assessed:

 1) Soil Characteristics: The manuscript does not provide information on the soil characteristics, such as pH, texture, and organic matter content, which could influence the fate and transport of PAHs in soil.

 2) PAH Source Apportionment: The study does not attempt to apportion the sources of PAHs in the soil, which could be an important aspect to understanding the contamination pathways and identifying effective mitigation strategies.

 Recommendations:

 1) Compare with existing literature: Conduct a more comprehensive review of existing literature on PAH levels in soil and their health implications to contextualize the findings and identify knowledge gaps.

 2) Provide more context: Expand the introduction to provide a more comprehensive overview of the environmental and health impacts of PAHs, as well as the current state of knowledge on this topic in Nigeria.

 3) Expand the study area: Consider increasing the study area to include multiple cities or regions to enhance the generalizability of the findings.

 4) Refine the sampling strategy: Develop a more systematic sampling strategy to minimize bias and ensure a representative sample.

 5) Provide more analytical method details: Include more information on the performance of the analytical method, such as the limit of detection, limit of quantitation, and precision.

 6) Enhance data analysis and presentation: Use more visual aids to present the results and facilitate easier interpretation and comparison of the data.

 7) Explore health implications further: Discuss the potential health risks associated with PAH exposure and explore ways to mitigate these risks, including policy recommendations and public health interventions.

 8) Enhance data interpretation and discussion: Expand the discussion to explore the implications of the findings, potential sources of PAHs contamination, and potential strategies for mitigation and remediation.

 9) Consider additional analyses: Consider conducting additional analyses, such as principal component analysis (PCA) or cluster analysis, to explore the relationships between different PAHs compounds and their potential sources.

 10) Include a section on limitations: Acknowledge the limitations of the study, including potential sources of bias, and discuss how these limitations may have impacted the findings.

 11) Consider including a section on policy implications: Discuss how the findings of this study can inform policy decisions related to environmental regulation, public health, and sustainable development in Nigeria.

 12) Provide more information about the workshops: Describe the characteristics of the automobile repair workshops, including their size, location, and operations, to provide a better understanding of the context in which the study was conducted.

 13) Explore the feasibility of remediation strategies: Discuss potential remediation strategies that could be employed to mitigate the impact of PAHs contamination in soil around automobile repair workshops.

 Conclusion:

 In conclusion, the manuscript presents a valuable study on the contamination of soil with PAHs around automobile repair workshops in Nigeria. Although, the study has some strengths, it also has some weaknesses that need to be addressed. By revising the manuscript to address these weaknesses and incorporating additional information on soil characteristics and PAH source apportionment, the authors can strengthen the study's contributions to the field of environmental science and public health.

Is the work clearly and accurately presented and does it cite the current literature?

No

If applicable, is the statistical analysis and its interpretation appropriate?

No

Are all the source data underlying the results available to ensure full reproducibility?

No

Is the study design appropriate and is the work technically sound?

No

Are the conclusions drawn adequately supported by the results?

No

Are sufficient details of methods and analysis provided to allow replication by others?

No

Reviewer Expertise:

Environmental Science and Public Health, with a specific focus on Environmental Toxicology and Risk Assessment. The study intersects with my fields of Analytical Chemistry, Environmental Monitoring, and Occupational Health. My expertise lies in the application of advanced analytical techniques, such as gas chromatography-mass spectrometry (GC-MS), to detect and quantify PAHs in environmental samples. The study's methodology also involves the use of toxic equivalency factors (TEFs) and incremental lifetime cancer risk (ILCR) to assess the carcinogenic potency of PAHs and the associated health risks. Overall, my area of research is interdisciplinary, drawing on concepts and methods from environmental science, chemistry, toxicology, and public health to investigate the environmental and health impacts of anthropogenic activities in developing countries.

I confirm that I have read this submission and believe that I have an appropriate level of expertise to confirm that it is of an acceptable scientific standard, however I have significant reservations, as outlined above.

## References

[1] : Evaluation of physicochemical characteristics and health risk of polycyclic aromatic hydrocarbons in borehole waters around automobile workshops in Southeastern Nigeria. *Groundwater for Sustainable Development* .2021;14: 10.1016/j.gsd.2021.100615 10.1016/j.gsd.2021.100615

[2] : Evaluation of concentrations of trace metal(loid)s in indigenous crab species and human health risk implications. *Emerging Contaminants* .2022;8: 10.1016/j.emcon.2022.07.001 371-380 10.1016/j.emcon.2022.07.001

[3] : Evaluation and risk assessment of heavy metals in King tuber mushroom in the contest of COVID-19 pandemic lockdown in Sokoto state, Nigeria. *Kuwait Journal of Science* .2024;51(2) : 10.1016/j.kjs.2024.100193 10.1016/j.kjs.2024.100193

# Reviewer report: Reviewer response for version 3

**DOI:** 10.5256/f1000research.161209.r246069

**Published:** 2024-03-05

**Authors:** Daniel O. Omokpariola

**Affiliations:** 1 Nnamdi Azikiwe University, Awka, Anambra, Nigeria

The authors have made significant corrective measures in the manuscript.

 Thanks for your efforts and time to complete all queries and concerns.

Is the work clearly and accurately presented and does it cite the current literature?

Partly

If applicable, is the statistical analysis and its interpretation appropriate?

Yes

Are all the source data underlying the results available to ensure full reproducibility?

Partly

Is the study design appropriate and is the work technically sound?

Yes

Are the conclusions drawn adequately supported by the results?

No

Are sufficient details of methods and analysis provided to allow replication by others?

Yes

Reviewer Expertise:

Environmental chemistry and toxicology; risk audit and process management; analytical chemistry

I confirm that I have read this submission and believe that I have an appropriate level of expertise to confirm that it is of an acceptable scientific standard.

# Reviewer report: Reviewer response for version 2

**DOI:** 10.5256/f1000research.158493.r222362

**Published:** 2023-11-24

**Authors:** Daniel O. Omokpariola

**Affiliations:** 1 Nnamdi Azikiwe University, Awka, Anambra, Nigeria

Thank you for your revision as there has been some improvement but there are pending areas:

**Materials:**

 The authors did not provide the sampling period and year/regimen used, as stated earlier.

**Discussion:**

 What is the impact of PAHs distribution for source identification? Please consult Okechukwu *et al.* (2021 ^1^); Edori and Iyama (2019 ^2^); Shamilishvily *et al.* (2018 ^3^) and so on.

 The health implications were not stated in regard to adverse health effects from exposure to humans (mechanics) as stated.

 Your conclusion stated that "The value of summation of ILCR and HQs from ingestion, inhalation, and dermal contact with PAHs contaminated soil is higher than the permissible limit stipulated by the World Health Organization." - what is WHO standards? And how does it impact overall wellbeing of humans and the environment at large?

Is the work clearly and accurately presented and does it cite the current literature?

Partly

If applicable, is the statistical analysis and its interpretation appropriate?

Yes

Are all the source data underlying the results available to ensure full reproducibility?

Partly

Is the study design appropriate and is the work technically sound?

Yes

Are the conclusions drawn adequately supported by the results?

No

Are sufficient details of methods and analysis provided to allow replication by others?

Yes

Reviewer Expertise:

NA

I confirm that I have read this submission and believe that I have an appropriate level of expertise to confirm that it is of an acceptable scientific standard, however I have significant reservations, as outlined above.

## References

[1] : Pollution investigation and risk assessment of polycyclic aromatic hydrocarbons in soil and water from selected dumpsite locations in rivers and Bayelsa State, Nigeria. *Environ Anal Health Toxicol* .2021;36(4) : 10.5620/eaht.2021023 e2021023-0 10.5620/eaht.2021023 34711020 PMC8850164

[2] : Source Identification of Polycyclic Aromatic Hydrocarbons in Water at Point of Effluent Discharge into the New Calabar River, Port Harcourt, Rivers State, Nigeria. *International Journal of Environment and Climate Change* .2019; 10.9734/ijecc/2019/v9i630119 343-349 10.9734/ijecc/2019/v9i630119

[3] : Polycyclic aromatic hydrocarbon in urban soils of an Eastern European megalopolis: distribution, source identification and cancer risk evaluation. *Solid Earth* .2018;9(3) : 10.5194/se-9-669-2018 669-682 10.5194/se-9-669-2018

# Response

**Published:** 2024-01-09

**Authors:** Olusola Adesina

**Affiliations:** Chemical engineering, Afe- Babalola University, Ado-Ekiti, Ekiti State, Nigeria

Thank you for your revision as there has been some improvement but there are pending areas:

**Materials:**

 The authors did not provide the sampling period and year/regimen used, as stated earlier.

Response:

 The sampling has now been added.

**Discussion:**

 What is the impact of PAHs distribution for source identification? Please consult Okechukwu *et al.* (2021 ^1^ ); Edori and Iyama (2019 ^2^ ); Shamilishvily *et al.* (2018 ^3^ ) and so on.

Response:

 The objective of the work does not involve source identification. We are sure that littering of the spent engine oil is the source of PAHs from observed in the soil.

 The health implications were not stated in regard to adverse health effects from exposure to humans (mechanics) as stated.

Response:

 This has been stated in the manuscript and it is also applies to the Mechanics (This indicates the chances of developing cancer from inhalation of this soil are slim. The combination of ILCR from accidental ingestion, inhalation and dermal contact gives 1×10−3 and 9.8×10−5 , for adults and children respectively.)

 Your conclusion stated that "The value of summation of ILCR and HQs from ingestion, inhalation, and dermal contact with PAHs contaminated soil is higher than the permissible limit stipulated by the World Health Organization." - what is WHO standards? And how does it impact overall wellbeing of humans and the environment at large?

Response:

 The limit WHO standard has now the added which 1×10 ^−6^. This has been stated in the manuscript. ILCR greeter than 1×10 ^−6  ^implies high chances of developing cancer during the lifetime while HQ higher 1 indicates high carcinogenic risk.

# Reviewer report: Reviewer response for version 1

**DOI:** 10.5256/f1000research.147757.r214475

**Published:** 2023-10-27

**Authors:** Akanimo N. Ekanem

**Affiliations:** 1 Department of Chemistry, Akwa Ibom State University, Mkpat Enin, Akwa Ibom, Nigeria

1. The statement “Few or no studies are available on the level of PAHs around automobile repair workshops, especially in Sub-Saharan Africa countries such as Nigeria” is not actually true because there are a lot of recent data on the subject matter such as Ekanem *et al.* (2019), Abugu *et al.* (2023), Daniel *et al.* (2023) etc.
2. The methodology is not explicit. In the soil sampling section, the apparatus used, sampling method and time of sampling is not mentioned. In the sample pretreatment section, quantity of solvent used for extraction is not mentioned. The references in the methods section are old. The author should review literature for the latest references.
3. In the discussion section, the authors could have presented a comprehensive table showing the concentrations of PAHs in all the samples analysed before presenting the statistical table. This would have enabled the reader to have comprehensive information about the study area.
4. The authors have not been able to drive home their point so as to draw conclusions on the health implication of PAHs. After comparison with the WHO standards, the authors would have made a conclusion whether the soil has low, moderate or high contamination and make recommendations.

Is the work clearly and accurately presented and does it cite the current literature?

Partly

If applicable, is the statistical analysis and its interpretation appropriate?

Yes

Are all the source data underlying the results available to ensure full reproducibility?

Partly

Is the study design appropriate and is the work technically sound?

Partly

Are the conclusions drawn adequately supported by the results?

Partly

Are sufficient details of methods and analysis provided to allow replication by others?

No

Reviewer Expertise:

Environmental toxicologist. I mostly monitor organic pollutants in the environment and their health implications.

I confirm that I have read this submission and believe that I have an appropriate level of expertise to confirm that it is of an acceptable scientific standard, however I have significant reservations, as outlined above.

# Response

**Published:** 2023-11-02

**Authors:** Olusola Adesina

**Affiliations:** Chemical engineering, Afe- Babalola University, Ado-Ekiti, Ekiti State, Nigeria

The authors evaluated the health risk assessment of PAHs in automobile workshops in Ado Ekiti with a mandate to assess the influence in the location, as I have some assertions which are not correct or may need to be worked on:

 1. The last sentence of your introduction "Few or no studies are available... in sub-Saharan African countries" is not correct because a clear check on google scholar states that over 100 papers have been published and data are available in Nigeria and sub-Saharan countries. Remodifying that statement is advised.

Response:

 This has been remodified.

 2. The materials and methods are okay within the scope of the work.

 3. The results and discussion does not provide a relationship and impact of these PAHs components to cause health based impact. More discussion is needed in relation to previous works available.

Response:

 ILCR and HQ are the indexes used to show the contamination of PAHs

 4. The conclusion is not adequate, as there are no recommendation or summary of the study in tandem to adults to children influences and how these automobile workshops can work effectively and dispose waste oils.

Response:

 This has been modified in the revised manuscript.

 Thanks.

# Response

**Published:** 2023-11-02

**Authors:** Olusola Adesina

**Affiliations:** Chemical engineering, Afe- Babalola University, Ado-Ekiti, Ekiti State, Nigeria

1. The statement “Few or no studies are available on the level of PAHs around automobile repair workshops, especially in Sub-Saharan Africa countries such as Nigeria” is not actually true because there are a lot of recent data on the subject matter such as Ekanem *et al.* (2019), Abugu *et al.* (2023), Daniel *et al.* (2023) etc.

Response:

 This part has been deleted in the revised manuscript.

  

1. The methodology is not explicit. In the soil sampling section, the apparatus used, sampling method and time of sampling is not mentioned. In the sample pretreatment section, quantity of solvent used for extraction is not mentioned. The references in the methods section are old. The author should review literature for the latest references.

Response:

 Authors appreciate this comment apparatus used for sampling has now been included, quantity of solvent used has now been included in the revised manuscript. Authors want to state that standard method for extraction and analysis of PAHs were duly followed

  

1. The authors have not been able to drive home their point so as to draw conclusions on the health implication of PAHs. After comparison with the WHO standards, the authors would have made a conclusion whether the soil has low, moderate or high contamination and make recommendations.

Response:

 Authors have taken care of this in the revised manuscript.

# Reviewer report: Reviewer response for version 1

**DOI:** 10.5256/f1000research.147757.r191176

**Published:** 2023-08-08

**Authors:** Daniel O. Omokpariola

**Affiliations:** 1 Nnamdi Azikiwe University, Awka, Anambra, Nigeria

The authors evaluated the health risk assessment of PAHs in automobile workshops in Ado Ekiti with a mandate to assess the influence in the location, as I have some assertions which are not correct or may need to be worked on:

 1. The last sentence of your introduction "Few or no studies are available... in sub-Saharan African countries" is not correct because a clear check on google scholar states that over 100 papers have been published and data are available in Nigeria and sub-Saharan countries. Remodifying that statement is advised.

 2. The materials and methods are okay within the scope of the work.

 3. The results and discussion does not provide a relationship and impact of these PAHs components to cause health based impact. More discussion is needed in relation to previous works available.

 4. The conclusion is not adequate, as there are no recommendation or summary of the study in tandem to adults to children influences and how these automobile workshops can work effectively and dispose waste oils.

 Thanks.

Is the work clearly and accurately presented and does it cite the current literature?

Partly

If applicable, is the statistical analysis and its interpretation appropriate?

Yes

Are all the source data underlying the results available to ensure full reproducibility?

Partly

Is the study design appropriate and is the work technically sound?

Yes

Are the conclusions drawn adequately supported by the results?

No

Are sufficient details of methods and analysis provided to allow replication by others?

Yes

Reviewer Expertise:

NA

I confirm that I have read this submission and believe that I have an appropriate level of expertise to confirm that it is of an acceptable scientific standard, however I have significant reservations, as outlined above.
